# Psychometric investigation of the gamification Hexad user types scale with Brazilian Portuguese adolescents speakers

**DOI:** 10.1038/s41598-023-45544-y

**Published:** 2023-10-30

**Authors:** Ana Cláudia Guimarães Santos, Pedro Kenzo Muramatsu, Wilk Oliveira, Sivaldo Joaquim, Juho Hamari, Seiji Isotani

**Affiliations:** 1 Institute of Mathematics and Computer Science, University of São Paulo, São Carlos, Brazil; 2 Gamification Group, Faculty of Information Technology and Communication Sciences, Tampere University, Tampere, Finland; 3 Education Center, Federal University of Alagoas, Maceió, Brazil; 4 Harvard Graduate School of Education, Cambridge, United States

**Keywords:** Computer science, Information technology, Human behaviour

## Abstract

Gamification has been applied in different fields over the last years, impacting the development of technologies, services, and products towards a more game-like world. Albeit its success, some results indicated personal differences influencing the success of its application, leading to the creation of user models (e.g., Hexad), a way to represent user profiles in gamified environments. Even though a great effort has been made to create and discuss instruments to represent these user models, many of them lack investigation into their psychometric properties. At the same time, although gamification can be particularly interesting in engaging adolescents, few attempts analyzed instruments considering this population. Addressing this lack, in this study, we evaluated the psychometric properties of the gamification Hexad scale in Brazilian Portuguese considering a sample ($N = 110$) of adolescents aged between 13 and 16 years old. Through a quantitative study (i.e., confirmatory factor analysis and correlation), we analyzed the psychometric properties of the scale and the correlations between user types when using data collected from adolescents. Results demonstrated that the current version of the scale needs improvements to better assess the user types of the Brazilian adolescent population, especially regarding the Disruptor user type. Also, the user types presented fewer correlations when compared with the adult sample. At the same time that the results of this study can be used in the academy and industry as a source of measurement of user types from Brazilian adolescents, it also opens several possibilities for new studies in personalized gamification considering this population.

## Introduction

Gamification is the process in which services, activities, and systems are transfigured to promote similar motivational benefits as found in games^1,2^. With applications ranging from education^3–5^ to well-being^6–8^, gamification has been utilized as a strategy to promote engagement and improve users’ overall engagement levels^9^. During the initial stage of gamification as an independent research field, which has begun around 10 years ago^10^, research focused on assessing *whether* gamification’s effectiveness^2^. Following many works that demonstrated mostly positive outcomes, as well as some neutral and negative outcomes^10,11^, research then matured into exploring the underlying gamification design strategies^2^. For instance, the need of adapting gamified systems to the needs of an individual user (i.e. tailored gamification) has seen a significant rise in the influence of interpersonal differences on the perception of gamification^5,9,12^.

Previous research on this topic demonstrated that demographic factors (e.g. age and gender) or personality traits can significantly alter how the user perceives gamification elements^9^. Nonetheless, due to the lack of a dedicated theoretical model that accurately describes interpersonal preferences on gamification, only the application of the aforesaid factors might not create effective tailored gamified systems^13^. Throughout the years, researchers have worked on how certain characteristics may affect the users while using a gamified system, and therefore, how people could be grouped into different profiles according to these characteristics and motivations^14–16^. Players and users typologies are used to try to simplify the complexity of the users^17,18^, and the choice of the user typology is one of the main factors that can influence the user motivation in personalized gamified environments^13^. The Hexad, a user model introduced by Marczewski^19^, is shown to be the only empirically validated (albeit partially)^20^ created exclusively for gamification. The Hexad model is based on Self-Determination Theory^21^, which classifies human motivation as intrinsic (when three basic human psychological needs (i.e., competence, autonomy, and relatedness) are supported) and extrinsic (when the reason for doing something is not an interest in the activity itself)^22^. In the Hexad, the user types derived from the intrinsic motivations are the Achievers (motivated by mastery), Socialisers (appreciating social interaction), Philanthropists (motivated by purpose), and Free-Spirits (driven by exploration), while Players (driven by extrinsic rewards) are the one derived from extrinsic motivations. Disruptors (who like to trigger change) are not a user type derived from Self-Determination Theory, but rather from the observation of user behavior in online systems^19,20^.

To assess the user types from the Hexad model, Tondello et al.^23^ developed the Hexad scale, originally created in English with 24 noninvasive items. After that, the Hexad scale has been psychometrically investigated in a number of languages. Based on this first version, Akgün and Topal^24^, analyzed the psychometric properties of Hexad in Turkish, which ultimately removed two items from the original scale (one from Player and the other from the Disruptor sub-scale) after the items presented weak load values and a high error rate in the confirmatory factor analysis (CFA). In 2019, Tondello et al.^20^ conducted a new study validating the Hexad scale and presenting novel items. Even though they tried to validate the scale in different languages, they were able to only analyze the scale in English and Spanish. Moreover, they indicated the need to validate the refurbished version of Hexad in other languages. Considering this, Taşkın and Çakmak^25^ conducted another validation of the new version of the Hexad scale in Turkish. By using the Single-Translation Method and focusing on the meaning rather than verbatim, their study was the first to adapt this new version of the scale^20^. Krath and von Korflesch^26^ conducted the validation of the scale using German and English versions. In this study, they identified a need for improvement of both scales to achieve a better model fit.

Recently, Krath et al.^27^ conducted a new study trying to reduce the Hexad scale in a short version. They developed and validated a 12-item version finding good psychometric properties, discriminant and convergent validity, besides indicating that this new version can represent the 24-item version. Therefore, their study is an indication that in the future, short versions of the Hexad scale can be successfully developed. The psychometric investigation of the Hexad scale targeted towards a specific age group was reported in two studies that focused on adolescents. Ooge et al.^28^, investigated the psychometric properties of Hexad in Dutch and were not able to confirm the validity of the scale, demonstrating that Hexad should not be applied to adolescent Dutch speakers. Manzano-León et al.^29^, investigated the psychometric properties of Hexad Spanish and English. Differently from Ooge et al.^28^, they identified suitable results, with no interference observed in regard to gender (i.e., boys and girls comprehended the scale in the same way).

Brazil, a country with 211 million native Brazilian Portuguese speakers, has been an undoubtedly relevant setting for conducting gamification studies^4,5^. However, only 5.1% of the Brazilian population have adequate English comprehension skills^30^. Therefore, the original version of the Hexad scale would not enable the conduction of studies with a more representative sample. This fact generates a necessity for instruments developed, translated, and validated into Brazilian Portuguese, allowing members of industry, researchers, or other interested parties to use instruments that can be employed with confidence. Facing this problem, about a year ago, we conducted a study investigating the psychometric properties of the Hexad scale with Brazilians^31^. Overall, this study indicated that the Hexad is an oblique model (i.e., there are correlations between the sub-scales) and even though the Brazilian Portuguese version has good internal reliability, some items from Disruptor and Free Spirit sub-scales needed a revision to properly assess the user types.

Although this first study advanced the literature by providing a psychometric analysis of the Hexad scale in Brazilian Portuguese, at that time, 91% of the dataset was composed of data collected from adults (older than 20 years). Therefore, there is no indication in the current literature of how the Brazilian version of the Hexad scale behaves toward adolescents. Considering this lack in the prior study and the fact that some of the studies about the Hexad scale considered specific samples from adolescents^28,29^, we conducted this study ($N = 110$) to evaluate the psychometric properties of the Brazilian Portuguese version of the Hexad scale considering a sample completely formed by data collected from adolescents aged between 13 and 16 years old. We conducted a confirmatory factor analysis, investigating the psychometric properties of the scale as well as a Kendall’s $\tau$ test to investigate possible correlation between the user types. Results indicated that the current version of the scale needs improvements to better measure the user types of the Brazilian adolescent population. Moreover, the adolescent sample presented less correlations between the user types than the study with adults as well as more problematic items. Therefore, our results indicated that the current version of the Hexad scale might be more suitable to assess the user types from adults than the user types from adolescents, which implicates the necessity of reformulation or the creation of new items for this type of user. Our results contribute to both researchers and practitioners by presenting analyses of an instrument to identify the Brazilian adolescent user types and insights on how to improve the Hexad user type scale.

## Methods

This study had as its main goal to analyze the psychometric properties of the Brazilian Portuguese version of the Hexad scale with adolescents. Different from the first study^31^ that analyzed the Hexad scale in Brazilian Portuguese with participants from different age groups, this current study focused on one specific age group (i.e., adolescents). This new study was necessary considering that 91% of the dataset in the first study was composed of data collected from people older than 20 years. Therefore, the study results from Santos et al.^31^ might not be representative of youngest samples, considering the particularities teenagers have when compared with adults.

The dataset used in the study was provided by a teacher from a public school in Brazil and was supplied anonymously after the collection. In the first semester of 2022, the teacher collected the Hexad user types of 123 students using the scale that had the psychometric properties analyzed in Brazilian Portuguese before^31^. The teacher followed all the indications of the prior study, i.e., the use of the Hexad scale in a 7-point Likert scale, including an “attention-check” statement between the Hexad items. The Brazilian version of the Hexad scale consists of 24 items that were separately translated by two independent native speakers into Brazilian Portuguese from the original version, and each item was compared and assessed by an independent third native speaker^20^.

Besides the Hexad profile, the teacher also collected the gaming habits of the students, asking them if they play games and the frequency. The Hexad scale applied to the students and analyzed in this study can be seen in the study conducted by Santos et al.^31^ and in Supplementary Table S1. Informed consent was obtained from all participants and their legal guardian(s).

### Participants

From the 123 responses the dataset provided, 13 were discarded for having missed the attention-check item. Therefore, we analyzed data from 110 participants, with ages ranging from 13 to 16 years old (M $=$ 14.2, SD $=$ 0.68). Most respondents (85%) reported that playing games was a habit, with 45% reporting playing daily, 35% playing rarely, 17% playing weekly, and 5% reporting not knowing how much they play. In accordance with the Brazilian National Health Council resolution number 510 published on April 7th, 2016, the participants of the study were not identified, and therefore, more demographic information about them was not provided to the authors.

### Statistical analysis

To evaluate the properties of the Hexad scale, we analyzed the (1) descriptive statistics, (2) internal reliability, (3) correlation between user types, and (4) factor analysis of the data. As the aim of the study was to assess the psychometric properties of a model (Hexad scale), according to Levine^32^, a Confirmatory Factor Analysis (CFA) is a more appropriate procedure in comparison with an Exploratory Factor Analysis (EFA).

We used the software IBM SPSS 27^33^ to conduct a Kolmogorov–Smirnov test (aiming to see if the data was parametric or non-parametric), to measure the descriptive statistics (i.e mean and standard deviation), the internal reliability (Cronbach’s $\alpha$), and the bivariate correlation coefficients (Kendall’s $\tau$) in the data obtained.

To conduct the CFA we used the software JASP 0.14.1^34^, utilizing structural equation modeling (SEM) with a robust Diagonally Weighted Least Squares (DWLS) method, which is a more accurate parameter estimate when the data are ordinal^35^. Considering that the Kolmogorov–Smirnov test indicated that our data were non-parametric, we used the robust option of the method as it is more suitable for analyzing data that does not follow a normal distribution^36^. To assess the model fit, we analyzed the Chi-Square ($\chi ^2$), the Relative Chi-square ($\chi ^2/df$), the Goodness of Fit Index (GFI), the Tucker–Lewis Index (TLI), the Comparative Fit Index (CFI), the Bentler–Bonett Normed Fit Index (NFI), the Standardized Root Mean Square Residuals (SRMR) and the Root Mean Square Error of Approximation (RMSEA) results, considering the goodness-of-fit indexes as $\chi ^2$ p $\ge$ 0.05; $\chi ^2/df$ $\le$ 3; GFI $\ge$ 0.95; TLI $\ge$ 0.95; CFI $\ge$ 0.95; NFI $\ge$ 0.95; SRMR $\le$ 0.08; and RMSEA $\le$ 0.06, following different recommendations^37–41^.

### Ethical statements

This study has been performed in accordance with the Brazilian National Health Council resolution number 510 published on April 7th, 2016, and with the relevant guidelines and regulations set by the Universities involved. Informed consent was obtained from all subjects and their legal guardian(s).

## Results

In this section, we present the results from the analyses of internal reliability, distribution of the Hexad user types, correlations presented between the user types, and the results from the confirmatory factor analysis.

### Internal reliability, correlations, and user type distribution

Initially, we analyzed the distribution of the data using the Kolmogorov–Smirnov test, which demonstrated that the scores were not normally distributed. After that, we measured the Means and the Standard Deviations in each Hexad sub-scale, which results can be seen in Table 1. We also measured the internal reliability of the data using Cronbach’s $\alpha$, which results are shown in Table 1. Overall, the reliability scores were acceptable ($\alpha$ $\ge$ 0.70), except for the Disruptor and Free Spirit sub-scales. Prior studies that analyzed the Hexad scale^20,25,26,28,31^ have also found similar results ($\alpha$ $\le$ 0.70) for these sub-scales. Finally, we measured the bivariate correlation coefficients between the user types’ scores, which results are shown in Table 1. Since the user type scores were non-parametric, as recommended by Wohlin^42^, we measured the bivariate correlation coefficients between each Hexad user type using Kendall’s $\tau$.

As reported in Table 1, the higher average scores were from the Player and Achiever sub-scales, results that are partially similar to prior studies of the Hexad scale^20,23,26,31^. The lower average scores were from Disruptors, which is the same result of all the studies that analyzed the Hexad scale^20,25,26,28,29,31^. We also calculated the dominant user types (i.e., the strongest tendency^13,31,43^) of each respondent, which distribution results were: Player $=$ 31%, Achiever $=$ 30%, Philanthropist $=$ 16%, Socialiser $=$ 12%, Free Spirit $=$ 8%, and Disruptor $=$ 3%. Most of the respondents (70%) presented more than one dominant user type, with Achiever and Player being the dominant user types of 61% of the respondents (14 students having both dominant user types).

**Table 1:** Descriptive analysis, internal reliability, bivariate correlation coefficients (Kendall’s $\tau$), and significance between each Hexad user type and all others. $N = 110$. UT: User type; M: mean score; SD: standard deviation; $\alpha$: Cronbach’s Alpha; A: Achiever; D: Disruptor; F: Free Spirit; P: Philanthropist; R: Player; S: Socialiser. **Correlation is significant at the 0.01 level (2-tailed). *Correlation is significant at the 0.05 level (2-tailed).

UT	M	SD	$\alpha$	A	D	F	P	R
A	23.14	5.103	0.750					
D	13.39	4.946	0.527	0.052				
F	21.65	4.042	0.489	0.244**	0.079			
P	21.23	5.154	0.761	0.203**	− 0.189**	0.255**		
R	23.12	5.048	0.742	0.323**	0.193**	0.281**	0.162*	
S	20.08	5.567	0.775	0.132	0.039	0.107	0.399**	0.172*

### Confirmatory factor analysis

**Figure 1:**
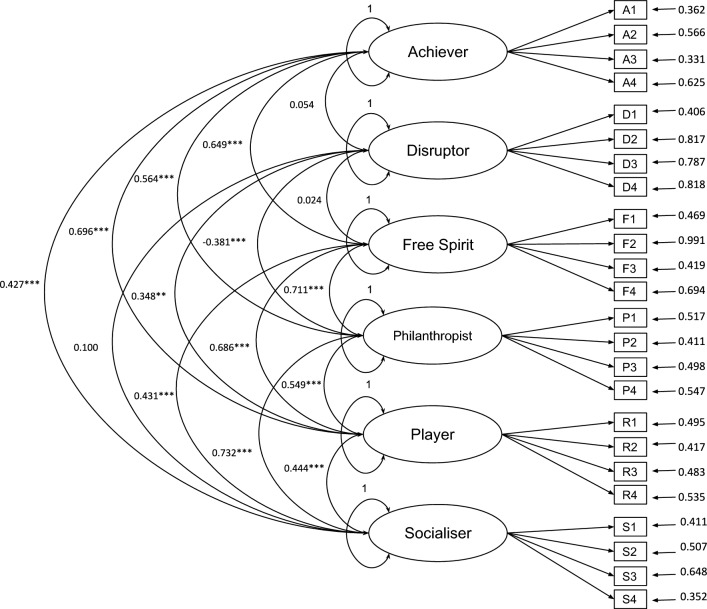
Path model with correlations between the factors. The ellipses represent the factors and the rectangles represent the items of the scale. ***$p < 0.001$; **$p< * < 0.005$. The variance in each factor is defined in 1 by JASP^34^. All parameters were freely estimated in the analysis.

Considering the results of the CFA that assessed the psychometric properties of the Hexad scale in Brazilian Portuguese with a majority adult sample^31^, and also prior studies about the Hexad scale^24,25,29^, we conducted the CFA considering the Hexad model as an oblique model (i.e., considering the factors as correlated). In this analysis, the six Hexad user types were modeled as latent variables correlated with each other, the 24 survey items were modeled as observed variables and the four items associated with each user type were modeled as reflections of the respective latent variable. Figure 1 presents the path model.

To evaluate the goodness of fit of the model, following Kline’s^44^ suggestion, we initially measured the chi-squared test $\chi ^2$ and the RMSEA. The Chi-squared test did not support the evidence for a good model fit ($\chi ^2_{237} = 347.422, \, p \le 0.001$). However, the Chi-squared test is sensitive to the sample size, normally rejecting the model fit when large samples are used and not discriminating good fitting models and poor fitting models when small samples are used^37^. Thus, we calculated the $\chi ^2/df = 1.46$, which indicated a good model fit^37,45^. The RMSEA $=$ 0.065 (CI $=$ [0.050, 0.080]), the CFI $=$ 0.969, the Tucker–Lewis Index (TLI) $=$ 0.964, the NFI $=$ 0.909, and the GFI $=$ 0.960, indicated a well-accepted fitted model^37,39,40^. However, the SRMR = 0.093 was slightly higher than the indicated^37^.

Table 2 present the factor loadings for each of the Hexad survey items. Based on the factor loadings and considering that Cronbach’s $\alpha$ may be misleading due to its tendency to underestimate reliability^46^, we also measured the composite reliability (CR) of each Hexad sub-scale. The CR is formulated through structural equation modeling and is equivalent to coefficient omega^47^, being a good option to measure reliability. The results found could be considered acceptable (CR $\ge$ 0.7) for all the factors except the Disruptor and Free Spirit sub-scale which results were slightly below the acceptable (Achiever $=$ 0.816; Disruptor $=$ 0.606; Free Spirit $=$ 0.640; Philanthropist $=$ 0.804; Player $=$ 0.811; and Socialiser $=$ 0.811).

**Table 2:** Factor loadings. $N = 110$. UT: User types/factors; I: Items; SE: standard errors; CR: critical ratios; CI: Confidence interval; $\lambda$: standardized $\lambda$ $\lambda$ $\ge$; A: Achiever; D: Disruptor; F: Free Spirit; P: Philanthropist; R: Player; S: Socialiser.

UT	I	SE	Z-value	CI		$\lambda$
				5%	95%	
**A**	**A1**	0.053	15.048	0.695	0.903	**0.799**
	**A2**	0.070	9.430	0.522	0.796	**0**.**659**
	**A3**	0.057	14.321	0.706	0.930	**0**.**818**
	**A4**	0.104	5.868	0.408	0.817	**0**.**612**
**D**	**D1**	0.079	9.769	0.616	0.926	**0**.**771**
	**D2**	0.098	4.365	0.235	0.619	0.427
	**D3**	0.083	5.551	0.299	0.625	0.462
	**D4**	0.093	4.603	0.245	0.609	0.427
**F**	**F1**	0.065	11.166	0.601	0.857	**0**.**729**
	**F2**	0.087	1.080	-0.076	0.264	0.094
	**F3**	0.064	11.986	0.638	0.887	**0**.**762**
	**F4**	0.084	6.607	0.389	0.717	**0**.**553**
**P**	**P1**	0.058	12.041	0.582	0.808	**0**.**695**
	**P2**	0.052	14.874	0.666	0.869	**0**.**767**
	**P3**	0.057	12.410	0.596	0.820	**0**.**708**
	**P4**	0.062	10.940	0.552	0.794	**0**.**673**
**R**	**R1**	0.055	12.847	0.602	0.819	**0**.**710**
	**R2**	0.054	14.162	0.658	0.870	**0**.**764**
	**R3**	0.051	14.061	0.619	0.819	**0**.**719**
	**R4**	0.073	9.371	0.539	0.825	**0**.**682**
**S**	**S1**	0.048	15.978	0.673	0.861	**0**.**767**
	**S2**	0.055	12.659	0.593	0.811	**0**.**702**
	**S3**	0.065	9.060	0.465	0.721	**0**.**593**
	**S4**	0.045	17.959	0.717	0.893	**0**.**805**

λ values ≥ 0.500 are in [bold].

## Discussion

In this study, we focused on analyzing the psychometric properties of the Hexad user types scale in Brazilian Portuguese considering an adolescent sample. To the best of our knowledge, this is the first study to conduct analysis of the Brazilian version of the scale considering an adolescent sample and the third overall that limited the age of the participants. Following other studies that analyzed the properties of the Hexad scale in other languages, we carried out a reliability analysis, a correlation analysis, and a confirmatory factor analysis (CFA) considering the Hexad as an oblique model. The CFA indicated problems with the Disruptor and Free Spirit sub-scales.

Our results indicated significant correlations between the user types, which are expected considering the fact that the underlying motivations of the user types are also related^20^. Similar results were found in the prior study with the adult Brazilian sample^31^, however, while the study with the adult Brazilian sample indicated significant correlations between all the user types, in this study the significant correlations were presented by 10 of the 15 correlations analyzed. This might be an indication that the older the user, the more difficult it would be to determine one specific dominant user type. Even though it is common in studies of the gamification field the definition of one dominant user type for the user when considering the Hexad^13,48^, this result corroborates the importance of the definition of the user profile as a combination of the different user types. About the correlations, similar to the study from the adult sample, the strongest correlation happened between Socialisers and Philanthropists (0.399**). Philanthropists and Socialisers have presented a correlation in several studies about the Hexad scale^20,25,26,29,31^ and this correlation can be explained by the fact that both user types are interested in social interaction, with Socialisers interested in the interaction itself and Philanthropists interested in interaction for altruistic purposes^20^. In this study with the adolescent sample, the second strongest correlation happened between Achievers and Players (0.323**). A possible explanation of the correlation between Achievers and Players, which was also found in prior research about the Hexad scale^20,25,26,29,31^, can be the fact that both users are motivated by achievement, with Players focusing on extrinsic rewards and Achievers focusing on competence.

In the CFA, we followed the studies conducted by Akgün and Topal^24^, Taşkın and Çakmak^25^, Manzano-León et al.^29^, and Santos et al.^31^ (i.e., our previous study), that indicated the factors should be modeled as correlated in the analysis. Therefore, we modeled the analysis considering the user types are correlated with each other (i.e. considering the Hexad as an oblique model). Overall, our study presented a good fit model ($\chi ^2/df = 1.46$, RMSEA $=$ 0.065, CFI $=$ 0.969, TLI $=$ 0.964, NFI $=$ 0.909, GFI $=$ 0.960, and SRMR $=$ 0.093), however, the factor loadings of the Disruptor (items 2, 3, and 4) and Free Spirit (item 2) sub-scales indicated problems with items ($\lambda \, \le \,0.5$). The items D3 and D4 were the same items that presented $\lambda$ $\le$ 0.5 in the study with the Brazilian adult sample^31^ when modeling the factors as correlated in the analysis. Therefore, overall when modeling the Hexad as an oblique model, the scale is more problematic with adolescents than adults. However, the items D2 and F2 also presented $\lambda$ $\le$ 0.5 in the previous study with the adult sample, when modeling the factors as non-correlated (i.e. considering the Hexad as an orthogonal model). We understand that this is an indication that the translations of items D1, D2, D3, and F4 into Brazilian Portuguese are not effective and should be reviewed regardless of the age of the user.

The worst $\lambda$ value presented in this study was from item F2. When considering the analyses that were made considering the Hexad as an oblique model (i.e., modeling the analysis without correlation between the factors), the study conducted by Ooge et al.^28^ with adolescents also indicated problems with the Free Spirit sub-scale. They indicated that to achieve a better factor structure, it would be necessary to remove all the Free Spirit sub-scale while assessing the Hexad user types of adolescents. This would implicate a simplification of the theoretical bases of the model when considering adolescent users. However, we understand that this problem is not correlated with the age of the respondents, since the study of Tondello et al.^20^ indicated that the item F2 presented a low factor loading, and suggested that this item would probably fit better with another user type. We believe that to properly assess the Free Spirit user type not only from adolescents but overall, it would be necessary to develop new items for this sub-scale.

Regarding the problems presented by the Disruptor sub-scale, the lowest $\lambda$ values were from D2 and D4. We understand that a possible explanation for the results of item D2 was the use of the Latin expression “status quo”. The other two studies that have analyzed the psychometric properties of the scale with an adolescent sample^28,29^, have explained to the respondents its meaning before the survey application, and other studies have replaced the expression with another expression in the validation language^20,25^. We understand that there is a necessity for reformulation of this item to improve the measurement of this user type. However, considering that most of the items of the Disruptor sub-scale have presented a low factor loading in this study, the problem might be with the user type itself. Since the launching of the model, several studies have presented negative results with the Disruptors (e.g., Disruptors presenting more negative results than the other user types^49,50^ and Disruptors performing worse in gamified applications when compared to the other user types^51^). Also, most of the studies that applied the Hexad scale considering different fields, have indicated that the lowest scores happen in the Disruptor sub-scale (e.g.,^50–54^). We understand that these results are an indication that a further investigation of this user type is necessary to understand more about the user type itself and also to learn how people of different ages present its characteristics.

Nowadays, Brazil has more than 200 million inhabitants and most of them cannot speak English fluently^30^. Therefore, it is essential (as a short-term alternative) to provide suitable instruments in Brazilian Portuguese. Considering the field of gamification, the Hexad scale is the most indicated instrument to assess user types in gamified systems and even though can be considered a recent instrument, it has already been evaluated in six different languages (i.e., English^20,26^, Spanish^20,29^, Turkish^25^, Dutch^28^, German^26^, and Portuguese^31^). However, most of these evaluations did not concentrate on analyzing the scale considering adolescents. Therefore, little is known regarding the scale’s effectiveness in certain populations. Overall, our results indicated that the Brazilian Portuguese version of the Hexad scale is useful for assessing the user types of adolescents, however, considering the problems with the Disruptor sub-scale, we understand that this user type is not being properly assessed when using the current version. Therefore, our study corroborates prior research^28^ when indicating that a specific version of the Hexad scale might be necessary to fully assess the Hexad user types of adolescents. The use of an evaluated scale is an important advance in future research involving this population and also in the development of gamified environments by industry. Therefore, the evaluation presented in this study may benefit several future studies with adolescents in Brazil.

## Limitations and opportunities for future studies

Our study has some limitations considering the following aspects: (1) sample, (2) age of participants, and (3) data from only one Portuguese-speaking country. Regarding the sample, the dataset was from one unique school, therefore, the results here presented might not be the same when analyzing a more diverse sample. Moreover, the dataset was provided to the authors without the possibility of collecting more information about the participants, which prevented us from analyzing a bigger sample or indicating possible correlations between the user types and demographic characteristics. Considering the dataset provided, differently from the first study, we were not able to analyze whether the instrument could be used regardless of gender. Therefore, we have no evidence about how gender (or other variables e.g., age and educational level) can influence the psychometric properties of the Brazilian version of the Hexad scale with adolescents. Also, all the participants were from 13-16 years old, therefore, the scale might not be applicable to people younger than 13 years old. Considering that the sample analyzed in the prior study of the Brazilian Portuguese version of the Hexad scale was majority composed of people older than 20 years, there is no evidence that the scale can measure well the user types from people aged between 17 and 20 years. Finally, the version analyzed in this study was the translated scale to Brazilian Portuguese, however, this translation cannot be the most suitable instrument to measure the Hexad user types in other countries that also have Portuguese as the official language (e.g. Portugal, Angola, Mozambique).

Based on these limitations and the study results, there are some possible studies that can be carried out in the future. Even though the Brazilian Portuguese version of the Hexad scale has been analyzed in two studies with two different samples, the scale has not yet been analyzed with children. Future studies can tackle this challenge generating results that could be very useful to designers and researchers in the development of gamified settings for them. In this study, the items D2, D3, D4, and F2 did not reach the expected factor loading values. Also, the same items have presented problems in the prior study with a Brazilian adult sample. We believe future studies should propose new translations for these items or new items to measure the Disruptor and Free Spirit sub-scales. These improvements seemed to be necessary for better measurement of the Hexad user types. Finally, considering that nine different countries have Portuguese as the official language, with each of them presenting language variances and cultural differences, future studies should adapt the Brazilian Portuguese scale to other Portuguese-speaking nations, expanding its use in Portuguese.

## Conclusion

Gamification is a recent research topic and user models that identify how users might behave in gamified systems are a current challenge in the field. Even though some instruments have been created to evaluate user profiles, most of them have not been analyzed in several widely spoken languages. Moreover, few studies have considered specific age groups (e.g., adolescents) when analyzing these instruments. In this study, we investigated the psychometric properties of the Hexad scale in Brazilian Portuguese considering an adolescent sample. Our results demonstrated that five of the six Hexad user types can be well measured with the current version and indicated the items that need improvement. Moreover, the results here presented are an indication that the current version of the Hexad scale is more appropriate for adult samples than for teenagers, leading to the need for an updated version for this population. In the future, we intend to conduct new studies about the scale using younger samples and also adapt and create new items for the user types that presented low factor loading in this study. Our study extends the literature about the Hexad scale at the same time provides some insights on a research agenda for the field, that can be filled by new studies considering samples from different languages. Having an appropriate scale to measure the Hexad user types can help designers and the industry in the creation and personalization of several types of gamified systems, therefore, the current study represents a research contribution to the field of personalized gamified systems for adolescents.

### Supplementary Information

Supplementary Information 1.

Supplementary Information 2.
