# Supplementary material for: Psychometric investigation of the gamification Hexad user types scale with Brazilian Portuguese adolescents speakers
Source: Sci Rep. 2023 Oct 30;13:18645. doi: 10.1038/s41598-023-45544-y (PMC10616289; doi:10.1038/s41598-023-45544-y)
Supplement: Supplementary file 2 — Supplementary Information 2. [file 41598_2023_45544_MOESM2_ESM.pdf]

		English items	Brazilian Portuguese items
**Philanthropist**	P1	It makes me happy if I am able to help others.	Sinto-me feliz se sou capaz de ajudar os outros.
	P2	I like helping others to orient themselves in new situations.	Gosto de ajudar os outros a se orientarem em situações novas.
	P3	I like sharing my knowledge.	Gosto de compartilhar meu conhecimento com os outros.
	P4	The well-being of others is important to me.	O bem-estar dos demais é importante para mim.
**Socialiser**	S1	Interacting with others is important to me.	Interagir com os demais é importante para mim.
	S2	I like being part of a team.	Gosto de fazer parte de uma equipe.
	S3	It is important to me to feel like I am part of a community.	É importante para mim sentir que faço parte de uma comunidade.
	S4	I enjoy group activities.	Gosto de atividades em grupo.
**Free Spirit**	F1	It is important to me to follow my own path.	É importante para mim seguir meu próprio caminho.
	F2*	I often let my curiosity guide me.	Frequentemente deixo-me guiar pela curiosidade.
	F3	Being independent is important to me.	Ser independente é importante para mim.
	F4	Opportunities for self-expression are important to me.	Considero importantes as oportunidades para expressar a mim mesmo.
**Achiever**	A1	I like defeating obstacles.	Gosto de superar obstáculos.
	A2	I like mastering difficult tasks.	Gosto de dominar tarefas difíceis.
	A3	It is important to me to continuously improve my skills.	É importante para mim aprimorar continuamente as minhas habilidades.
	A4	I enjoy emerging victorious out of difficult circumstances.	Gosto de sair vitorioso de circunstâncias difíceis.
**Player**	R1	I like competitions where a prize can be won.	Gosto de competições em que possa ganhar prêmios.
	R2	Rewards are a great way to motivate me.	Recompensas são uma ótima forma de me motivar.
	R3	Return of investment is important to me.	Retorno de investimento é importante para mim.
	R4	If the reward is sufficient I will put in the effort.	Se a recompensa for suficiente, farei o esforço.
**Disruptor**	D1	I like to provoke.	Gosto de provocar.
	D2*	I like to question the status quo.	Gosto de questionar o status quo.
	D3*	I see myself as a rebel.	Vejo-me como um rebelde.
	D4*	I dislike following rules.	Não gosto de seguir regras.

Table S1. The original scale in English and the scale in Brazilian Portuguese. *: items that presented $\lambda \leq 0.500$ in the CFA. Instructions to use the scale: to use the scale, ask the respondents on a 7-point Likert scale to rate how well each item describes them. Present the items randomly to guarantee that the respondent is not able to identify the items that are from the same sub-scale. To guarantee that the respondents are reading all the statements before providing an answer, include an "attention-check" item in the middle of the Hexad items. To calculate the user type, add the scores the user presented in each sub-scale. The user type is formed by the six scores from the scale, with the highest score as the dominant user type. Instructions to use the scale (in Brazilian Portuguese): *Para usar a escala, peça aos respondentes que avaliem em uma escala Likert de 7 pontos, o quão bem cada item da escala Hexad os descreve. Apresente os itens de forma aleatória, de modo que o respondente não consiga identificar quais são os itens de cada perfil de usuário. Para garantir que os respondentes estejam lendo inteiramente os itens, inclua um "item de atenção" entre os itens da escala Hexad. Para definir o perfil de usuário do respondente, some a pontuação de cada sub-scala. O perfil de usuário é formado pelas seis pontuações, com a maior pontuação sendo o perfil de usuário dominante.*
